# Supplementary material for: Multi-ancestry sleep-by-SNP interaction analysis in 126,926 individuals reveals lipid loci stratified by sleep duration
Source: Nat Commun. 2019 Nov 12;10:5121. doi: 10.1038/s41467-019-12958-0 (PMC6851116; doi:10.1038/s41467-019-12958-0)
Supplement: Supplementary file 18 — Reporting Summary [file 41467_2019_12958_MOESM18_ESM.pdf]

natureresearch

Corresponding author(s): Raymond Noordam, PhD
Professor Susan Redline MD, MPH

Last updated by author(s): Sep 29, 2019

Reporting Summary

Nature Research wishes to improve the reproducibility of the work that we publish. This form provides structure for consistency and transparency in reporting. For further information on Nature Research policies, see Authors & Referees and the Editorial Policy Checklist .

Statistics

For all statistical analyses, confirm that the following items are present in the figure legend, table legend, main text, or Methods section.

n/a	Confirmed	
☐	☒	The exact sample size (*n*) for each experimental group/condition, given as a discrete number and unit of measurement
☐	☒	A statement on whether measurements were taken from distinct samples or whether the same sample was measured repeatedly
☐	☒	The statistical test(s) used AND whether they are one- or two-sided<br>*Only common tests should be described solely by name; describe more complex techniques in the Methods section.*
☐	☒	A description of all covariates tested
☐	☒	A description of any assumptions or corrections, such as tests of normality and adjustment for multiple comparisons
☐	☒	A full description of the statistical parameters including central tendency (e.g. means) or other basic estimates (e.g. regression coefficient) AND variation (e.g. standard deviation) or associated estimates of uncertainty (e.g. confidence intervals)
☐	☒	For null hypothesis testing, the test statistic (e.g. *F*, *t*, *r*) with confidence intervals, effect sizes, degrees of freedom and *P* value noted<br>*Give P values as exact values whenever suitable.*
☒	☐	For Bayesian analysis, information on the choice of priors and Markov chain Monte Carlo settings
☐	☒	For hierarchical and complex designs, identification of the appropriate level for tests and full reporting of outcomes
☐	☒	Estimates of effect sizes (e.g. Cohen's *d*, Pearson's *r*), indicating how they were calculated

Our web collection on statistics for biologists contains articles on many of the points above.

Software and code

Policy information about availability of computer code

Data collection	No software was used.
Data analysis	Cohort-level statistical analyses were performed using different (open source) statistical programs (see supplementary tables 2 and 4). To obtain robust estimates of covariance matrices and standard errors, studies with unrelated participants used R packages such as either sandwich or ProbABEL. Studies including related individuals used either generalized estimating equations (R package geepack) or linear mixed models (GenABEL, MMAP, or R package sandwich). Central cleaning was performed using EasyQC (https://www.uni-regensburg.de/medizin/epidemiologie-praeventivmedizin/genetische-epidemiologie/software/) as implemented in R (v 3.15). Meta-analyses were performed using METAL.

For manuscripts utilizing custom algorithms or software that are central to the research but not yet described in published literature, software must be made available to editors/reviewers. We strongly encourage code deposition in a community repository (e.g. GitHub). See the Nature Research guidelines for submitting code & software for further information.

Data

Policy information about availability of data

All manuscripts must include a data availability statement. This statement should provide the following information, where applicable:

- Accession codes, unique identifiers, or web links for publicly available datasets
- A list of figures that have associated raw data
- A description of any restrictions on data availability

Summary results files from both the trans-ancestry and European meta-analyses are available to the public via the CHARGE (Cohorts for Heart and Ageing Research in Genomics Epidemiology) dbGaP summary site (phs000930).

Field-specific reporting

Please select the one below that is the best fit for your research. If you are not sure, read the appropriate sections before making your selection.

- [x] Life sciences
- [ ] Behavioural & social sciences
- [ ] Ecological, evolutionary & environmental sciences

For a reference copy of the document with all sections, see nature.com/documents/nr-reporting-summary-flat.pdf

Life sciences study design

All studies must disclose on these points even when the disclosure is negative.

Sample size	We attempted to maximize the sample size to actively earch for cohorts with data available to contribute to the present analysis.
Data exclusions	Discovery and replication analyses comprised men and women between the age of 18 and 80 years, and were conducted separately for the different contributing (self-defined) ancestry groups, including: European, African, Asian, Hispanic, and Brazilian (discovery analysis only).
Replication	We performed a replication study in independent cohorts. All SNPs selected in Stage 1 for replication were evaluated in the interaction model in up to 18 cohorts contributing data from 20 study groups totalling up to 64,469 individuals (Supplementary Tables 4-6).
Randomization	This study was not a randomized clinical trail.
Blinding	This study was not a randomized clinical trial; no blinding was performed.

Reporting for specific materials, systems and methods

We require information from authors about some types of materials, experimental systems and methods used in many studies. Here, indicate whether each material, system or method listed is relevant to your study. If you are not sure if a list item applies to your research, read the appropriate section before selecting a response.

Materials & experimental systems

n/a	Involved in the study	
x		Antibodies
x		Eukaryotic cell lines
x		Palaeontology
x		Animals and other organisms
	x	Human research participants
x		Clinical data

Methods

n/a	Involved in the study	
x		ChIP-seq
x		Flow cytometry
x		MRI-based neuroimaging

Human research participants

Policy information about studies involving human research participants

Population characteristics	Discovery analyses were performed in up to 62,457 individuals (40,041 European-ancestry, 14,908 African-ancestry, 4,460 Hispanic-ancestry, 2,379 Asian-ancestry, and 669 Brazilian/mixed-ancestry individuals) from 21 studies spanning 5 different ancestry groups (Supplementary Tables 1 and 2; Supplementary Data 1). Of the total discovery analysis, 13,046 (20.9%) individuals were classified as short sleepers and 12,317 (19.7%) individuals as long sleepers. Replication analyses were performed in up to 64,469 individuals (47,612 European-ancestry, 12,578 Hispanic-ancestry, 3,133 Asian-ancestry, and 1,146 African-ancestry individuals) from 19 studies spanning 4 different ancestry groups (Supplementary Table 3 and 4; Supplementary Data 2). Of the total replication analysis, 12,952 (20.1%) individuals were classified as short sleepers and 12,834 (19.9%) individuals as long sleepers.
Recruitment	Cohort-specific recruitment is different for every cohort; detailed description is provided in the supplementary information.
Ethics oversight	The present work was approved by the Institutional Review Board of Washington University in St. Louis and complies with all relevant ethical regulations. Each participating study obtained written informed consent from all participants and received approval from the appropriate local institutional review boards.

Note that full information on the approval of the study protocol must also be provided in the manuscript.
