# Supplementary material for: Multi-ancestry sleep-by-SNP interaction analysis in 126,926 individuals reveals lipid loci stratified by sleep duration
Source: Nat Commun. 2019 Nov 12;10:5121. doi: 10.1038/s41467-019-12958-0 (PMC6851116; doi:10.1038/s41467-019-12958-0)
Supplement: Supplementary file 19 — Description of Additional Supplementary Files [file 41467_2019_12958_MOESM19_ESM.pdf]

Title: Supplementary Data 1
Description: Trait Distribution in Stage 1 studies

Title: Supplementary Data 2
Description: Trait Distribution in Stage 2 studies

Title: Supplementary Data 3
Description: Replicated lead SNPs from known regions for blood lipid levels in multi-ancestry cohorts - Long Total Sleep Time

Title: Supplementary Data 4
Description: Replicated leads SNPs from known regions for blood lipid levels in multi-ancestry cohorts - Short Total Sleep Time

Title: Supplementary Data 5
Description: Interaction effects of known SNPs with either LTST or STST

Title: Supplementary Data 6
Description: Replicated novel lead SNPs for blood lipid levels in multi-ancestry cohorts - Long Total Sleep Time

Title: Supplementary Data 7
Description: Replicated novel lead SNPs for blood lipid levels in multi-ancestry cohorts - Short Total Sleep Time

Title: Supplementary Data 8
Description: Replicated novellead SNPs for blood lipid levels in European ancestry cohorts - Long Total Sleep Time

Title: Supplementary Data 9
Description: Replicated novellead SNPs for blood lipid levels in European ancestry cohorts - Short Total Sleep Time

Title: Supplementary Data 10
Description: SNP main effects stratified by exposure to LTST in the multi-ancestry meta-analyses based on the discovery cohorts

Title: Supplementary Data 11
Description: SNP main effects stratified by exposure to STST in the multi-ancestry meta-analyses based on the discovery cohorts

Title: Supplementary Data 12
Description: Look-up of newly identified loci on different questionnaire-based sleep phenotypes in the UK Biobank

Title: Supplementary Data 13
Description: Look-up of newly identified loci on different accelerometer-based sleep phenotypes in the UK Biobank

Title: Supplementary Data 14
Description: gene look-up in the GWAS catalog (p<5e-8), italic font indicates sleep-related trait (p>5e-8)

Title: Supplementary Data 15
Description: Differential expression data of novel SNPs in different tissues from the GTEx database
